# Lack of cross-neutralization by SARS patient sera towards SARS-CoV-2

**DOI:** 10.1080/22221751.2020.1761267

**Published:** 2020-05-08

**Authors:** Danielle E. Anderson, Chee Wah Tan, Wan Ni Chia, Barnaby E. Young, Martin Linster, JennyG. H. Low, Yee-Joo Tan, Mark I.-C. Chen, Gavin J. D. Smith, Yee Sin Leo, David C. Lye, Lin-Fa Wang

**Affiliations:** a Duke-NUS Medical School, Singapore, Singapore; b National Center for Infectious Diseases, Singapore, Singapore; c Singapore General Hospital, Singapore, Singapore; d National University of Singapore, Singapore, Singapore

**Keywords:** SARS, COVID-19, antibody, cross-neutralization, SARS-CoV-2

## Abstract

Despite initial findings indicating that SARS-CoV and SARS-CoV-2 are genetically related belonging to the same virus species and that the two viruses used the same entry receptor, angiotensin-converting enzyme 2 (ACE2), our data demonstrated that there is no detectable cross-neutralization by SARS patient sera against SARS-CoV-2. We also found that there are significant levels of neutralizing antibodies in recovered SARS patients 9–17 years after initial infection. These findings will be of significant use in guiding the development of serologic tests, formulating convalescent plasma therapy strategies, and assessing the longevity of protective immunity for SARS-related coronaviruses in general as well as vaccine efficacy.

The outbreak of coronavirus disease 2019 (COVID-19) caused by SARS-CoV-2 started in Wuhan, China and has spread globally with sustained human-to-human transmission outside China. Singapore in Southeast Asia reported the highest number of 5050 confirmed cases as of 18 April 2020. Singapore also had the third highest number of confirmed SARS cases in 2003 [1,2]. As SARS-CoV and SARS-CoV-2 are genetically closely related and belong to the same viral species, *SARS related coronavirus* (SARSr-CoV) [3], it is important to understand the cross reaction/neutralization dynamics in affected patients, especially in regions heavily affected by both viruses, such as Singapore. This information is important for several reasons. First, virus-specific serological tests will play an important role in retrospective contact tracing, in monitoring potential asymptomatic infections such as in children and in tracing the origin and potential intermediate host(s). Second, pre-existing cross-reactive antibodies in a given population may play a role in disease transmission and severity as antibody-dependent enhancement is known for coronaviruses including SARS-CoV [4]. Third, the possibility of using SARS convalescent human plasma for treatment of COVID-19 patients needs to be assessed urgently for nations like Singapore. Lastly, such information may also shed light on the longevity of protective immunity for SARSr-CoV in general and on the development of effective vaccines for SARS-CoV-2.

For this study, convalescent sera obtained from 12 SARS survivors were used. As shown in Table 1, the collection times vary from <1 year to 17 years after SARS-CoV infection in 2003. The COVID-19 sera were collected from 24 January to 7 February 2020 from 7 patients admitted at Singapore General Hospital and the National Centre for Infectious Diseases. These sera represent different time points post onset of clinical symptoms.

**Table 1.** Summary of serological test results.

Serum group	Sample/Case ID	Years/days post symptom onset	Virus neutralization test^a^		ELISA with N protein^b^	
			SARS-CoV	SARS-CoV-2	SARS-CoV	hSARS-CoV-2
**SARS**	S1	< 1 year	1:80	<1:20	1.55	1.40
	S2	< 1 year	1:40	<1:20	2.23	2.34
	S3	< 1 year	1:40	<1:20	1.51	1.39
	S4	< 1 year	1:40	<1:20	1.58	1.44
	S5	< 1 year	1:80	<1:20	1.79	1.56
	S6	< 1 year	1:80	<1:20	1.65	1.55
	S7	< 1 year	1:160	<1:20	1.83	2.08
	S8	9 years	1:320	<1:20	0.25	0.27
	S9	9 years	1:320	<1:20	0.37	0.40
	S9	14 years	1:160	<1:20	0.56	0.53
	S10	17 years	1:160	<1:20	0.40	0.47
	S11	17 years	1:80	<1:20	0.95	0.98
	S12	17 years	1:20	<1:20	0.14	0.13
**Negative control**	N1	NA	<1:20	<1:20	0.06	0.08
	N2	NA	<1:20	<1:20	0.05	0.08
	N3	NA	<1:20	<1:20	0.06	0.09
	N4	NA	<1:20	<1:20	0.06	0.09
**COVID-19**	C1	6 days	<1:20	<1:20	0.03	0.05
	C1	20 days	<1:20	1:80	0.66	0.71
	C2	4 days	<1:20	<1:20	0.08	0.05
	C2	12 days	<1:20	1:80	2.11	2.28
	C2	18 days	1:20	1:80	2.08	2.42
	C3	4 days	<1:20	1:160	2.02	2.20
	C3	15 days	1:40	1:320	2.24	2.32
	C4	9 days	<1:20	<1:20	0.09	0.09
	C5	11 days	1:20	1:320	2.11	2.34
	C6	7 days	<1:20	<1:20	0.05	0.05
	C7	10 days	1:20	1:160	0.91	0.89

^a^Average Neutralization titers determined from three separate experiments.

^b^Average specific OD readings normalized by dividing the OD readings from human sera by OD readings for each antigen from anti-His monoclonal antibody as both N proteins were expressed with an His-tag.

Two serological test platforms, virus neutralization test (VNT) and Enzyme-linked immunosorbent assay (ELISA), were employed in this study. For VNT, we used a SARS-CoV-2 strain isolated from a COVID-19 patient in Singapore. This patient was confirmed positive by PCR on 22 January 2020 and live virus was isolated by inoculating Vero-E6 cells with an oral-nasal swab in our ABSL3 facility. The complete genome sequence is deposited in GISAID under the strain name BetaCoV/Singapore/2/2020 (Accession ID EPI_ISL_406973). VNT was conducted by preincubating 50 µl of diluted virus (5X10^3^ TCID50/ml) with 50 µl of diluted serum (or plasma) at 37°C for 90 min, using a two-fold dilution starting at 1:20. The mixture was then added to Vero E6 cells virus (10^4^ cells/well) in a 96-well plate, incubated at 37°C for 60 min, and washed with culture medium. The result is read after incubation at 37°C for 4–5 days. Neutralization antibody titres are expressed as the highest serum dilution which shows 100% inhibition of cytopathic effect (CPE).

For ELISA, recombinant nucleocapsid protein (N) from SARS-CoV and SARS-CoV-2, respectively, was expressed in HEK293T cells using the pcDNA3.1 vector system and purified using an affinity column using previously published method [5]. ELISA wells were coated with 100 ng protein per well and sera at 1:200 dilution, followed by HRP-conjugated goat anti-human antibody (Santa Cruz) at 1:2,000.

The spike (S) proteins of the two viruses are 75% identical at their amino acid sequence level, and the same level of identity also exists for the key receptor binding domain (RBD) [3]. Despite this genetic relatedness and the fact that both viruses use the same cell entry receptor, angiotensin-converting enzyme 2 (ACE2) [3], our data demonstrated that the level of cross-neutralization between SARS-CoV and SARS-CoV-2 is limited (Table 1). Some COVID-19 patient sera show low levels of neutralizing activity against SARS-CoV, but no neutralization of SARS-CoV-2 by SARS patient sera. This is different from previous findings indicating cross-neutralization by hyperimmune horse anti-SARS-CoV serum on SARS-CoV-2 virus [3] or by SARS patient sera or rabbit hyperimmune sera on pseudovirus carrying the spike protein of SARS-CoV-2 [6]. The particular horse anti-SARS-CoV hyperimmune serum used by Zhou et al. [3] was known to have a 10-fold greater neutralizing antibody level and binding to more S protein epitopes than most other human and animal sera [7], hence not surprising to see a low level of cross-neutralization with SARS-CoV-2. Similarly, pseudoviurs-based VNT is usually more sensitive than live virus-based VNT [8], which might be responsible for the cross-neutralization observed in the Hoffman et al. study [6].

On the other hand, there is significant cross-reactivity between the two N proteins, to the degree that it is almost impossible to differentiate between COVID-19 or SARS patient sera regardless of which N protein is used as ELISA antigen.

In conclusion, our data indicate that the cross-neutralization level of SARS survivors’ sera against SARS-CoV-2 is not sufficient for potential passive immunotherapy of COVID-19 patients. The strong cross-reactivity of N-directed antibodies proved the close relatedness of the two viruses and should be taken into consideration when developing serological tests and vaccine candidates. The potential impact of existing SARS-CoV antibodies on the severity of SARS-CoV-2 infection requires urgent investigation considering the high level of cross-reactive and largely non-neutralizing antibodies in SARS survivors. Finally, the finding of neutralizing antibodies in SARS survivors 9–17 years after the initiation infection is significant in the context of better understanding the longevity of SARSr-CoV protective immunity in general and vaccine development for SARS-CoV-2.
